# High-Fidelity Simulation to Assess Task Load Index and Performance: A Prospective Observational Study

**DOI:** 10.5152/TJAR.2022.21234

**Published:** 2022-08-01

**Authors:** Jérémy Favre-Félix, Mikhail Dziadzko, Christian Bauer, Antoine Duclos, Jean-Jacques Lehot, Thomas Rimmelé, Marc Lilot

**Affiliations:** 1 Lyonnais Center for Education by Simulation in Health, Claude Bernard Lyon 1 University, SAMSEI, Lyon, France; 2 Department of Anaesthesiology and Reanimation, Hospices Civils de Lyon, Croix Rousse Hospital, Lyon, France; 3 Department of Health Data, Hospices Civils de Lyon, Claude Bernard Lyon 1 University, France; 4 Research on Healthcare Performance (RESHAPE), Claude Bernard Lyon 1 University, Lyon, France; 5 Department of Anaesthesiology and Reanimation, Hospices Civils de Lyon, Pierre Wertheimer Hospital, Lyon, France; 6 Department of Anaesthesia and Reanimation, Hospices Civils de Lyon, Edouard Herriot Hospital, Claude Bernard Lyon 1 University, Lyon, France; 7 Department of Anaesthesia, Hospices Civils de Lyon, Woman Mother Child Hospital, Lyon, France

**Keywords:** Anaesthesia, clinical performance, high-fidelity simulation, Task Load Index

## Abstract

**Objective:**

The NASA Task Load Index is a questionnaire widely used in aviation. This index might help for attesting the quality of a scenario in high-fidelity simulation in healthcare. The main purpose of this study was to observe whether NASA Task Load Index for critical care-simulated scenarios, designed for residents, was consistent with the literature. The second purpose was to describe relationships between NASA Task Load Index, performance and generated stress during high-fidelity simulation.

**Methods:**

All residents in anaesthesia and intensive care undergoing high-fidelity simulation were included. The primary endpoint was the task load generated by each scenario assessed by NASA Task Load Index. Based on the literature, the NASA Task Load Index scores between 39 and 61 were considered an acceptable level. Stress level (Visual Analogue Scale) and specific technical and non-technical skills performances (Team Emergency Assessment Measure) were also assessed.

**Results:**

Totally 53 residents actively participated in 1 of 10 different scenarios, between June and December 2017. The median NASA Task Load Index score of scenarios was 61 [48-65]. Five scenarios generated acceptable task load levels. There was no association between the NASA Task Load Index score and technical or non-technical skills performance scores, but an association between NASA Task Load Index and the stress level (rho = 4.7, *P*  = .001) was observed.

**Conclusion:**

Simulation scenarios generate different task loads for residents; the task load was deemed acceptable for half of the scenarios. The NASA Task Load Index could be considered as a tool to assess the pedagogic adequacy of scenarios. Scenario and generated stress level, but not task load, can modify residents’ performance during simulation. This should be considered when planning normative simulation.

**Main Points**

- No association between task load score and performance has been observed.
- An association between task load and stress level of participants has been observed.
- Task load of scenario should be considered when planning normative simulation.

## Introduction

High-fidelity simulation (HFS) is used as an effective teaching method to enhance the acquisition of required competencies in anaesthesiology and intensive care.^1,2^ By taking part in HFS scenarios, residents are actively involved in relevant critical situations.^3^ High-fidelity simulation increases stress, mobilizing a level of mental and physical resources which may be expressed as the task load.^4,5^ The relationship between stress and efficient memorization was reported to look like an inverted U shape function.^6^ Too little stress would not help optimal learning, whereas too much stress would prevent the student from memorizing relevant information.^7^ Therefore, the scenario should be assessed for its potential task load generation and associated increase in stress level. Similarly, one may hypothesize that the optimal task load would be associated with higher performance, and the performance may be reduced in situations when the task load is too low or too high.^8-10^

Different behavioural and subjective metrics are used in operational environments (aviation, nuclear power plants, and medicine) to assess the task load of operators.^8-11^ The NASA Task Load Index (NASA-TLX), initially developed for flight training, is the most commonly applied tool to assess procedural workload in healthcare and has been specifically evaluated in the surgical field.^12^ This tool evaluates perceived task load immediately after the performed task through mental, emotional, and physical dimensions.

We hypothesized that the task load in anaesthesia and intensive care residents generated by the scenarios was consistent with the expected normal values reported in the literature. The main objective of this study was to explore the task load (with the NASA-TLX) of each scenario during HFS for anaesthesia and intensive care residents. Secondary objectives were to describe the relationships between NASA-TLX, scenario performance, and associated stress level.

## Methods

### Design

This observational prospective cohort study was conducted at the university medical simulation centre of Lyon (Lyon University, France). The study obtained approval from the Hospices Civil de Lyon institutional ethics committee (June 27, 2017) and has been pre-registered on clinicaltrial.gov (protocol ID: NCT03175484). Informed written consent was obtained from all enrolled participants. This research has been carried out in accordance with The Code of Ethics of the World Medical Association (Declaration of Helsinki).

### Population and S imulation S etting

This study involved all residents in anaesthesia and intensive care undergoing HFS sessions between June and December 2017. No exclusion criterion was applied. The scenarios simulated crisis situations occurring in the operating room, in the intensive care unit, or during intra-hospital patient transport. Scenarios were developed by instructors based on national guidelines. High-fidelity simulation sessions followed the standard repetitive sequences of briefing, scenario, and debriefing.

### Experimental P rotocol

The demographic data of participants at the beginning of the HFS session were collected (age, gender, previous HFS participation, and post-graduate year). We used the NASA-TLX questionnaire to assess the task load of each participant during each HFS scenario. NASA-Task Load Index score (0 point: no task load and 100 points: maximal task load) included 6 dimensions (mental demand, physical demand, temporal demand, performance, effort, and frustration). A detailed description and the questionnaires used are reported in Appendix A. As suggested in the literature, the NASA-TLX score was considered consistent with those values if situated between 25th and 75th percentile (39 < NASA-TLX < 61).^13^ We considered those NASA-TLX values as “acceptable.”

**Figure 1.**
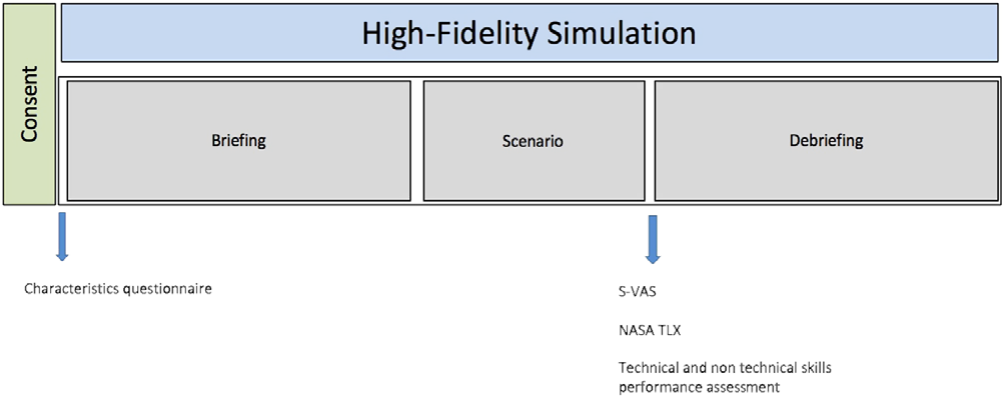
Timeline during high-fidelity simulation. S-VAS, Visual Analogue Scale for Stress; TLX, Task Load Index.

We measured the individual quantitative stress level using a Visual Analogue Scale for stress translated on a 100-mm numeric scale for stress (0: no stress and 100: maximal stress) immediately after each scenario. We also measured both technical performance, using specific technical skills scoring grid (previously described,^14^ 0: low performance and 100: maximal performance) and non-technical skills performance by the Team Emergency Assessment Measure scale (0: low performance and 44: maximal performance).^15,16^ Two investigators (C.B., M.L.) independently evaluated the resident performance using video recording. The timeline of the study is presented in Figure 1.

### Endpoints

The primary endpoint was the assessment of individual NASA-TLX score for each scenario. Secondary endpoints were the stress level at the end of the scenario measured by the Visual Analogue Scale for stress and the technical and the Team Emergency Assessment Measure performance score.

### Statistical A nalysis

Continuous variables were described using median (25th-75th percentile) and were compared using the Wilcoxon or the Student’s *t* test as appropriate. The Kruskal–Wallis test was used for more than 2 group comparisons. A multivariate linear regression analysis was performed to explore the interaction between NASA-TLX, scenario, stress, and technical performance. The correlation between technical performance and non-technical performance skills scores was evaluated using Spearman’s (rho) correlation index. All tests were 2-tailed, and *P* < .05 was considered statistically significant. Statistical analysis was performed on a *per-protocol* basis using MedCalc software version 9.6.4.0 (MedCalc, Mariakerke, Belgium).

## Results

A total of 53 residents (median age: 25 years old (min-max: 23-35, 25th-75th [24-27]), mean age: 26 years old, standard deviation = 2; 21 (40%) females), came through HFS and involved in 1 of 10 different scenarios, were included and were analysed from June to December 2017. The median NASA-TLX score of the 10 scenarios was 61 [48-65]. Five scenarios (50%) generated NASA-TLX between 25th and 75th percentile (39-61 points), and 5 scenarios generated a higher task load level (>61 points). The median NASA-TLX score of the 53 residents for all scenarios was 61.7 [46.7-67.1], 16 participants (30%) had a score between 25th and 75th percentile (39-61 points), 8 (15%) had a lower score, and 29 (55%) had a higher score.

The median Visual Analogue Scale for stress was 43.0 [24.5-66.0], the median technical skills performance was 44.0 [34.0-52.5], and the median Team Emergency Assessment Measure score was 23.5 [20.0-28.0]. There was no age or gender difference observed in NASA-TLX and Visual Analogue Scale for stress and performance scores. Except for the Visual Analogue Scale for stress, NASA-TLX and performance scores were significantly different across performed scenarios (Table 1).

**Figure 2.**
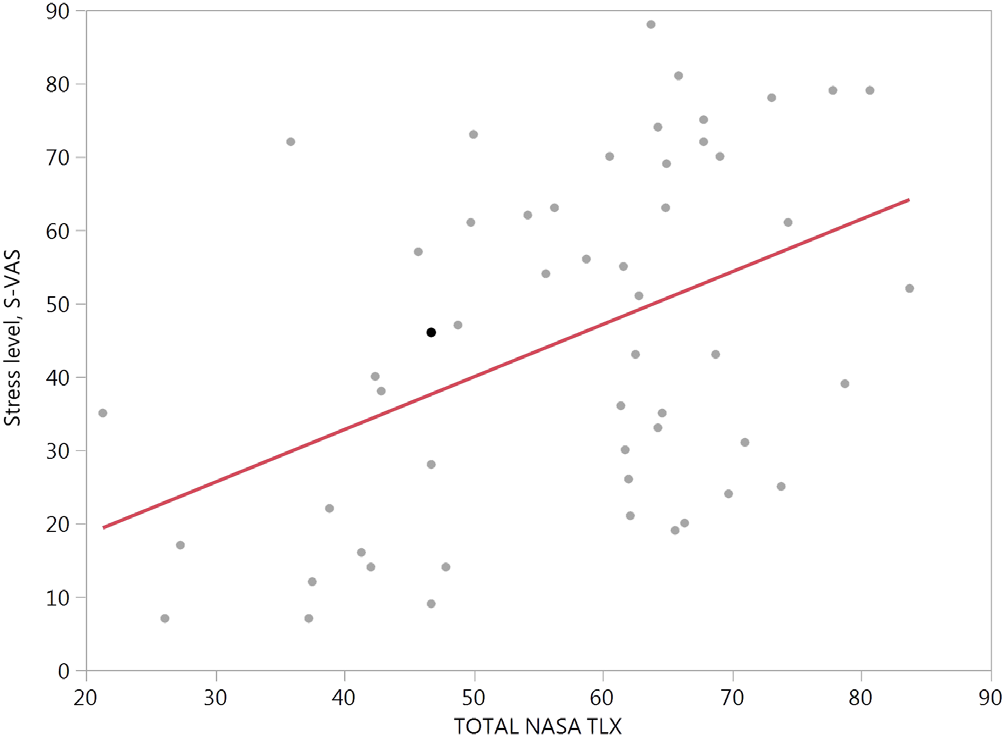
Bivariate fit of stress level and NASA Task Load IndeX score. Predicted Total NASA TLX = 44.685 + 0.279 × S-VAS, *P* = .001. S-VAS, Visual Analogue Scale for Stress; TLX, Task Load Index.

The technical skills performance was correlated with the non-technical skills performance (rho = 0.6; *P* < .001). In a bivariate fit analysis, NASA-TLX score was not associated with technical skills performance and Team Emergency Assessment Measure scores but was associated with stress level (4.7 points of increment for 1 point increment of NASA-TLX score, *P*  = .001, Figure 2).

An elevated Visual Analogue Scale for stress predicted decreased technical skills performance (−15 points decrement for each 10 points Visual Analogue Scale for stress increment, *P*  = .03). No significant association between Visual Analogue Scale for stress and the Team Emergency Assessment Measure score was observed.

In multivariate analysis, the Visual Analogue Scale for stress incurred by HFS (F ratio = 4.16, *P*  = .048) and the type of scenario (F ratio = 17.6, *P* < .0001) predicted technical skills performance. For the Team Emergency Assessment Measure performance, only the type of scenario (F ratio = 7.99, *P* < .0001) was predictive (Appendix B).

## Discussion

The task load level was assessed with the NASA-TLX score in 53 anaesthesia and intensive care residents undergoing HFS training. The median NASA-TLX score of the 10 scenarios was 61 [48 - 65]. Among the scenarios tested, one half generated an adapted task load and another half generated high task load level. Such scenarios are of great educational value but should be used with caution and may be suited to more experienced residents.

To the best of our knowledge, no study reporting a relationship between NASA-TLX and HFS performance scores in healthcare has been published. Different scales are available to measure task load. The subjective workload assessment technique uses 3 levels (low, medium, and high) for each of 3 dimensions of time load, mental effort load, and psychological stress. Overall workload allows the subjects to rate on a unidimensional scale from 0 to 100 points. Studies that compared all of these validated scales demonstrated the reliability and validity of the NASA-TLX in comparison to other workload measures,^17^ probably due to the 6 subscales which allow more precision in the task load assessment. Hart et al^18^ showed that NASA-TLX was the most used subjective scale to assess task load. Previous validation in the medical field has been published providing information to help interpret the obtained scores for each scenario.^13^ However, to the best of our knowledge, no study validated objective methods to assess task load in the medical field.

The use of task load assessment by NASA-TLX was first described in aviation. It was demonstrated that reducing the task load significantly improved performance in aircraft.^19^ In healthcare, an increasing number of studies focus on task load measurement since overload has been identified as a significant cause of errors.^20,21^ Increased response time during simulated crisis situations has also been reported with task overload.^22,23^ Residents might experience high task load during scenarios and this may harm the process of learning. By contrast, scenarios with too low task load might result in poor involvement. Task load analysis becomes important when the cognitive load theory is considered. This theory assumes that working memory capacity is limited. In some complex learning cases, reducing task load will help increase working memory capacity.^24^ In the present study, no extreme NASA-TLX scores (>77 points) were observed, a level reported as clear overload in the literature.^13^

An association between NASA-TLX and stress level was observed, while no association was observed with the technical or the non-technical performance. Those results suggest that NASA-TLX could be used as a marker of scenarios’ educational quality but not as a single tool to assess residents’ performance during HFS. Similar results were found in a surgical simulation study exploring NASA-TLX during laparoscopy,^25^ which might be explained by the fact that performance and task load are 2 separate dimensions of the scenario. High-fidelity simulation scenario might be perceived as a challenge with a need to mobilize new working resources, such as providing leadership during critical situations. These tasks will affect the task load without systematically affecting performance skills. The performance scores obtained during HFS result from multifactorial and complex factors that affect the participants before and during the scenario. The performance during the scenario is not a pedagogical objective of the HFS but rather a tool to achieve better further performance in a real setting (helped with the information provided during the debriefing). Thus, we might assume that performance during HFS is not, in this study, a valuable independent marker for the scenario’s educational quality. Moreover, the perceived performance is only one component of the 6 scales of NASA-TLX questionnaire and this might explain in parts why performance was not associated with NASA-TLX. The performance NASA-TLX subscale reflects the subjective self-evaluation of residents that could be influenced by several psychometric or emotional factors (self-efficacy feeling, fear of negative evaluation, social anxiety, and reaction of participants to the announcement of the end of the scenario).^5,26-28^ This auto-appreciation does not fit with the performance scale relying on specific objectives rated by investigators.

The association between task load and stress was previously described in surgery residents. NASA-Task Load Index was positively correlated with objective stress levels measured by sympathetic activity (heart rate and blood pressure).^29^ These changes may be explained by specific NASA-TLX subscales as frustration and temporal demand, which are the feelings of being overpassed that could increase stress levels.

This study has several limitations. The number of residents was 53 and could have precluded the inference of NASA-TLX and performance. Ten scenarios for 3 different post-graduate years were included offering variability that might have influenced the results. Moreover, post-graduate years of residency might influence the stress levels during HFS in complex and multifactorial ways explaining the unpredictability of that relationship.^7^ Then, as Hart et al^18^ reported previously, we noticed that the main limitation of the NASA-TLX is the interpretation of the score. Thresholds used in this study were given by the analysis of the vast amounts of data published in the medical field.^13^ This analysis, mainly in simulated endoscopic surgery and emergency room situations, did not match the setting for the participants (alone or in a team), and the performance did not impact any certification. All of these differences could have influenced the task load and it is very difficult to establish a universal and reliable threshold in the medical field. However, identifying the relative task load for each scenario might help instructors to identify the specific interest of the scenario in regard to the task load provided. Further studies are needed to assess the potential of error productions associated with perceived scenario-specific task load and to further evaluate the hypothetical difficulty of each scenario. There is a need for precision on the optimal NASA-TLX threshold to define locally what is an acceptable high or low workload, in order to use this tool effectively to enhance the pedagogical values of HFS. NASA-Task Load Index might be used as a tool to identify scenarios with outliers or marginal scores in order to select, upgrade, or adapt scenarios to the pedagogical objectives of HFS. The performance has been explored with specific technical skills evaluation grid and one non-technical skill evaluation grid. Although several studies have been reported to observe the difference in performance score (technical and non-technical skills), still some performance may not be covered by these performance grids.^30,31^ Therefore, one might suspect that the NASA-TLX explored some other information that could impact performance in a way that is not observed by a singular performance grid. Although no association between performance and NASA-TLX was observed here, a deeper exploration of task load level impact on performance should be further explored to confirm the lack of clear association.^32^

## Conclusion

To conclude, simulation scenarios generate different task loads in residents and NASA-TLX could be considered as an additional tool to help instructors to assess the pedagogic adequacy of HFS scenarios to learners. Scenario and generated stress level, but not task load, can modify residents’ performance during simulation. This should be considered when planning normative simulation.

## Figures and Tables

**Table:** 

**Source**	**Nparm**	**DFNum**	**DFDen**	**F Ratio**	*P*
**Total NASA TLX**	1	1	41	0.21	.65
**Scenarios**	9	9	41	17.65	<.0001
**S-VAS**	1	1	41	4.16	.045

**Table:** 

**Source**	**Nparm**	**DFNum**	**DFDen**	**F Ratio**	*P*
**Total NASA-TLX**	1	1	41	0.36	.55
**Scenarios**	9	9	41	7.99	<.0001
**S-VAS**	1	1	41	0.03	.86

**Table 1.** Description of Scenarios, Residents Involved, NASA-Task Load IndeX, Skills Performance and Stress Level Across High-Fidelity Simulation Scenarios. Values Are Median [IQR Range]

Topic of the Scenario	Summary of the Briefing	PGY	Male/Female (n/n)	NASA-TLX	Technical Performance	Non-technical Skills Performance	S-VAS
Announcement of a surgery side error	In the PACU, you receive the patient’s family to explain surgery side error	5, n = 4	2/2	46 [38-64]	41 [36-53]	20 [19-28]	40 [13-68]
Cardiogenic shock	You admit in the ICU a young man with acute hypotension, tachycardia, and moderate fever context	5, n = 4	2/2	64 [63-75]	56 [47-62]	33 [21-38]	41 [36-62]
Severe postpartum haemorrhagic shock	You are called for a woman with severe bleeding after delivery	5, n = 4	2/2	65 [61-70]	76 [47-62]	34 [29-37]	32 [22-61]
Cardiac arrest due to local anaesthetics toxicity	You are in charge of a patient under regional anaesthesia for a fracture fixation	5, n = 4	2/2	45 [40-70]	61 [55-67]	36 [34-37]	18 [14-64]
Acute neurological disorders due to local anaesthetics toxicity	You are in charge of a patient under regional anaesthesia for a fracture fixation	2, n = 4	4/0	64 [62-72]	31 [27-36]	20 [19-24]	67 [38-84]
Gas embolism	You are in charge of a patient with spinal surgery under general anaesthesia, when acute dyspnoea occurs	2, n = 4	4/0	53 [33-56]	39 [29-45]	21 [19-26]	58 [26-70]
Acute post reperfusion ventricular fibrillation	You are in charge of a patient for of acute lower limb ischaemia surgery	2, n = 5	3/2	62 [48-73]	36 [34-45]	28 [23-29]	47 [33-80]
Acute hypoxia due to selective intubation	You are called to transport a patient to the ICU after hyperbaric oxygenotherapy	1, n = 8	4/4	42 [29-46]	42 [41-44]	21 [20-23]	31 [ 9-39]
Tracheal tube obstruction occurring during an intra-hospital transport	You transport an intubated patient with pneumonia to the CT-scan	1, n = 8	4/4	60 [51-65]	42 [38-45]	17 [17-24]	53 [24-62]
Compressive pneumothorax occurring during an intra-hospital transport	You transport an intubated patient with chest trauma to the CT-scan	1, n = 8	5/3	68 [63-74]	48 [43-52]	26 [21-27]	53 [30-74]
Comparison test				Kruskal–Wallis, *P* =.0046	Kruskal–Wallis, *P* < .0001	Kruskal–Wallis, *P* < .0005	Kruskal–Wallis, *P* =.35

PGY, post-graduate year; TLX, Task Load IndeX; S-VAS, Visual Analogue Scale for Stress; PACU, post anaesthesia care unit; ICU, intensive care unit; CT, computed tomography.

## Appendix A: NASA-TLX questionnaire and detailed description

NASA-TLX score was calculated in 2 different steps dealing with 6 subscales (mental demand, physical demand, temporal demand, performance, effort, frustration).

First, each of the 6 subscales was quoted for its perceived importance in the workload in a visual analogue scale (VAS). Second, each subscale was associated in pairs with others (i.e., total of 15 pairs of subscales) and participant had to select for each pair, the subscale that influenced most of the workload. Then, each subscale was weighted with the VAS multiplied by the number of times the subscale was selected and divided by 15. The overall NASA-TLX score was calculated as the sum of each weighted subscale. The score was a number from 0 (task load minimal) to 100 (task load maximal).

## Appendix B: Multivariate Analysis Regression Model

B.1 Regression Model for Technical Performance Skill

Source, lists the fixed effects in the model; Nparm, number of parameters associated with the effect; DFNum, numerator degrees of freedom for the effect test; DFDen, denominator degrees of freedom for the effect test; F atio, computed F ratio for testing that the effect is 0; TLX, Task Load Index; S-VAS, Visual Analogue scale for Stress.

B.2 Regression Model for Non-technical Performance Skills

Source, lists the fixed effects in the model; Nparm, number of parameters associated with the effect; DFNum, numerator degrees of freedom for the effect test; DFDen, denominator degrees of freedom for the effect test; F ratio, computed F ratio for testing that the effect is 0; TLX, Task Load Index; S-VAS, Visual Analogue scale for Stress.
